# Novel technique of cholangioscopy-guided inwardly migrated stent retrieval

**DOI:** 10.1055/a-2127-5045

**Published:** 2023-08-21

**Authors:** Rami Reddy Yalaka, G. S. Sameer Kumar, Kondal Reddy Mogili, Chandan Kumar Kedarisetty, Raghavendra Babu Yalakanti

**Affiliations:** 1 Star Hospitals, Hyderabad, India; 2 Gleneagles Global Hospitals, Hyderabad, India

We present a case of a 54-year-old man who had undergone a liver transplant and developed a biliary anastomotic stricture. The patient underwent endoscopic retrograde cholangiopancreatography (ERCP) and stenting for the treatment of the condition. He visited our facility for a stent exchange. During the ERCP procedure, it was discovered that one of the stents had migrated inward ( Fig. 1 ). Despite multiple attempts using the biliary balloon, basket, and snare for stent extraction, all efforts were unsuccessful 1 2 3 4 . Consequently, we decided to perform cholangioscopy (SpyGlass DS; Boston Scientific, Marlborough, Massachusetts, USA), which revealed that the distal end of the stent was lodged in the wall of the distal common bile duct ( Video 1 ). However, the deep impaction made it impossible to employ the spy snare. Therefore, we opted to disimpact the stent 5 .

**Fig. 1:**
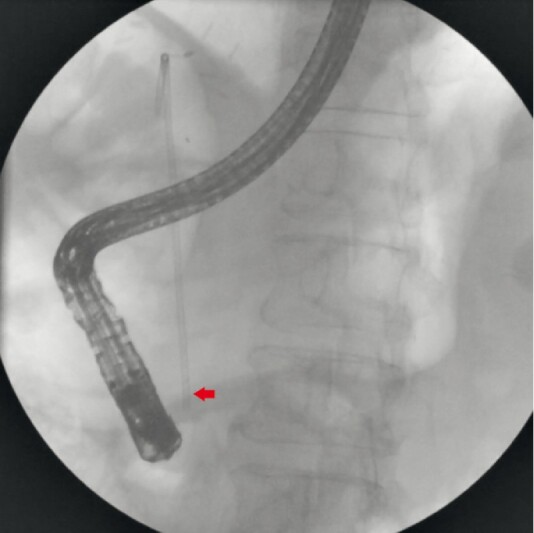
Fluoroscopic image showing inwardly migrated common bile duct stent. Impacted distal end of biliary stent (arrow).

**Video 1** Novel technique of cholangioscopy-guided inwardly migrated stent retrieval.


During cholangioscopy, we identified a side opening at the distal flange of the stent. We inserted a guidewire through the side hole and into the stent lumen. Subsequently, we exchanged the cholangioscope with a sphincterotome (Ultratome; Boston Scientific) over the guidewire, engaging it into the side hole of the stent. By pushing the stent inward, we successfully disimpacted the distal end. Once disimpaction was achieved, we replaced the sphincterotome with a routine snare over the guidewire. Under fluoroscopy guidance, we captured the distal end of the stent with the snare. Finally, the guidewire, stent, and snare complex were retrieved along with the scope ( Fig. 2–5 ). We then placed two new plastic stents across the stricture.

**Fig. 2:**
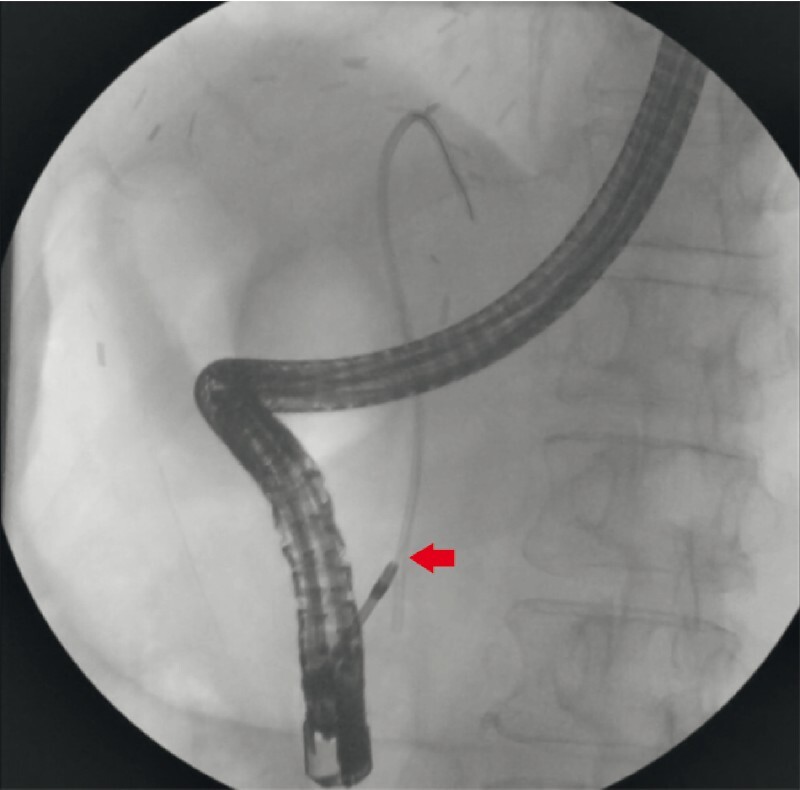
Fluoroscopic image showing cholangioscopy-guided cannulation of migrated biliary stent (arrow).

**Fig. 3:**
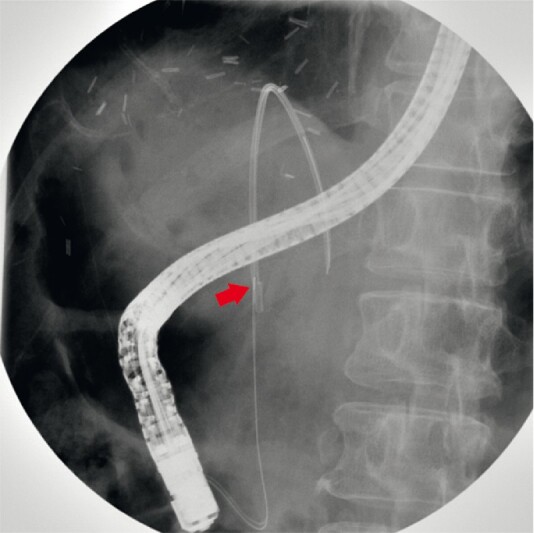
Fluoroscopic image showing disimpacted biliary stent in mid common bile duct with the help of sphincterotome (arrow).

**Fig. 4:**
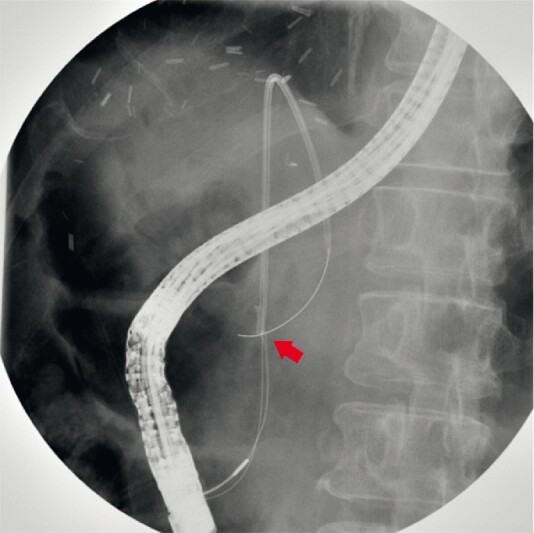
Fluoroscopic image showing ensnaring the distal end of the migrated biliary stent over the guidewire (arrow).

**Fig. 5:**
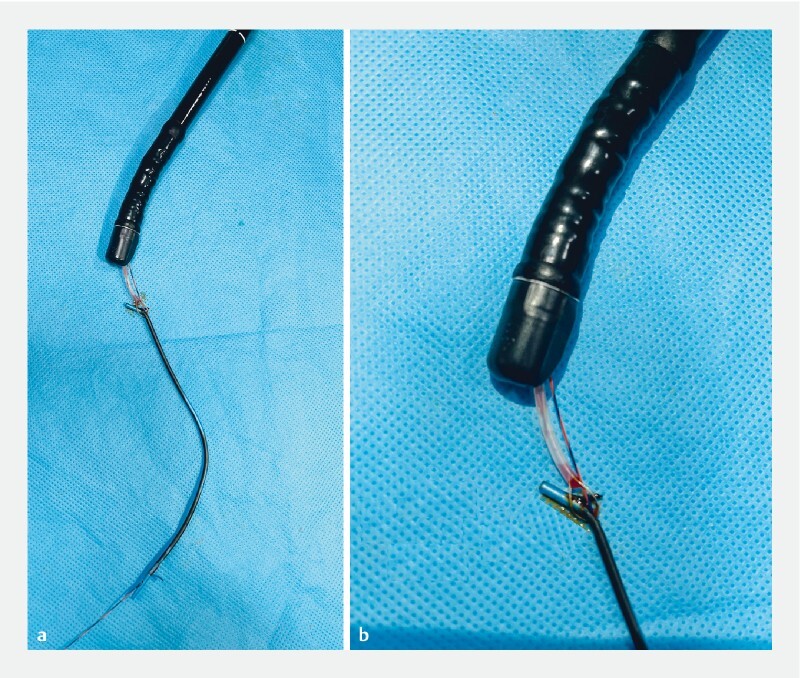
In vitro stent, snare, and guidewire complex. **a** With scope. **b** Closer view.

To the best of our knowledge, this is the first case report highlighting the successful use of cholangioscope-guided guidewire cannulation through the side hole and the utilization of a sphincterotome and routine snare for the retrieval of a migrated stent, thus obviating the need for surgery.

Endoscopy_UCTN_Code_CPL_1AK_2AD

## Notes

Endoscopy E-Videos

https://eref.thieme.de/e-videos
*E-Videos* is an open access online section of the journal *Endoscopy* , reporting on interesting cases and new techniques in gastroenterological endoscopy. All papers include a high-quality video and are published with a Creative Commons CC-BY license. Endoscopy E-Videos qualify for HINARI discounts and waivers and eligibility is automatically checked during the submission process. We grant 100% waivers to articles whose corresponding authors are based in Group A countries and 50% waivers to those who are based in Group B countries as classified by Research4Life (see: https://www.research4life.org/access/eligibility/ ). This section has its own submission website at https://mc.manuscriptcentral.com/e-videos
